# Toxicity of *Beauveria bassiana* to *Bactrocera dorsalis* and effects on its natural predators

**DOI:** 10.3389/fmicb.2024.1362089

**Published:** 2024-05-02

**Authors:** Xin-lian Li, Jing-jing Zhang, Dou-dou Li, Xin-yan Cai, Yi-xiang Qi, Yong-yue Lu

**Affiliations:** Department of Entomology, College of Plant Protection, South China Agricultural University, Guangzhou, China

**Keywords:** biological control, entomopathogenic fungi, environmental factors, germination, insecticide, integrated pest management, Moniliaceae, Tephritidae

## Abstract

Entomopathogenic fungi (EPF) are economical and environmentally friendly, forming an essential part of integrated pest management strategies. We screened six strains of *Beauveria bassiana* (B1–B6) (Hypocreales: Cordycipitaceae), of which B4 was the most virulent to *Bactrocera dorsalis* (Hendel) (Diptera: Tephritidae). We further assessed the biological characteristics of strain B4 and the environmental factors influencing its ability to infect *B. dorsalis*. We also evaluated the effects of B4 on two of the natural predators of *B. dorsalis*. We found that strain B4 was the most virulent to 3rd instar larvae, pupae, and adult *B. dorsalis*, causing mortality rates of 52.67, 61.33, and 90.67%, respectively. B4 was not toxic to *B. dorsalis* eggs. The optimum B4 effects on *B. dorsalis* were achieved at a relative humidity of 91–100% and a temperature of 25°C. Among the six insecticides commonly used for *B. dorsalis* control, 1.8% abamectin emulsifiable concentrate had the strongest inhibitory effect on B4 strain germination. B4 spraying affected both natural enemies (*Amblyseius cucumeris* and *Anastatus japonicus*), reducing the number of *A. cucumeris* and killing *A. japonicus* adults. We found a valuable strain of EPF (B4) that is virulent against many life stages of *B. dorsalis* and has great potential for the biological control of *B. dorsalis*. We also provide an important theoretical and practical base for developing a potential fungicide to control *B. dorsalis*.

## 1 Introduction

*Bactrocera dorsalis* (Hendel) (Diptera: Tephritidae) is one of the most economically serious invasive pests worldwide (Vontas et al., 2011). It is widely distributed in tropical and subtropical regions, damaging a wide variety of fruits and fleshy vegetables (Liu et al., 2019; Li et al., 2021). This fruit fly mainly lays eggs inside fruits and vegetables as an adult, and the larvae subsequently feed on the fruits and cause damage. It is precisely this concealed method of damage by fruit flies that makes their prevention and control challenging (Wakil et al., 2022). To combat the harmful fruit fly, growers largely rely on chemical insecticides. However, although effective, they have many adverse effects with long-term use, including resistance problems, non-target effects, and harm to humans and the environment (Jin et al., 2011a; Hsu et al., 2012; Khan et al., 2014; Chen et al., 2019; Awan et al., 2021).

Due to the range of issues associated with the use of chemical insecticides, it is essential to develop more environmentally friendly fruit fly control methods (Daane and Johnson, 2010). Biological control methods based on natural predators, parasitoids, and entomopathogenic fungi (EPF) are regarded as safe substitutes for chemical pesticides (Dorta et al., 2020). For a long time, exploring the use of pathogenic microorganisms as a strategy against pests has been a focal point of research (Hajek and St Leger, 1994).

*Beauveria bassiana* (Hypocreales: Cordycipitaceae) is a pathogenic microorganism commonly used in insect control. It has been applied to control several pests, achieving significant social, economic, and ecological benefits (Butt and Copping, 2000; Cai, 2003; McKinnon et al., 2017). Several studies have evaluated the impact of *B. bassiana* on fly species (Diptera: Tephritidae), including *Ceratitis capitata* (Quesada-Moraga et al., 2006; Ekesi et al., 2010), *Bactrocera oleae* (Sookar et al., 2008), *Bactrocera zonata* (Usman et al., 2021; Wakil et al., 2022), and *Bactrocera cucurbitae* (Onsongo et al., 2022). However, a limited number of studies have focused on the use of *B. bassiana* against *B. dorsalis,* and most studies on the pathogenicity of *B. bassiana* against *B. dorsalis* have been conducted in the laboratory (Pan et al., 2008; Zhang et al., 2010; Pan and Zhai, 2015; Wang et al., 2021; Menzler-Hokkanen et al., 2022).

We selected the B4 strain, which was the most virulent to all life stages of *B. dorsalis*, from the six *B. bassiana* strains, and evaluated the effects of environmental factors (temperature, relative humidity, and relative soil water content), different concentrations, and chemical reagents on the virulence of *B. bassiana* (B4) on *B. dorsalis* and two of its natural enemies. Our research provides an important theoretical and practical basis for developing targeted *B. bassiana* agents and insecticide applications to effectively control different life stages of *B. dorsalis* in the field.

## 2 Materials and methods

### 2.1 *Beauveria bassiana* strain cultures

We obtained six strains of *B. bassiana* (B1–B6). Strain B1 was provided by the Laboratory of Fungal Pesticide Creation, Institute of Agricultural Environment and Sustainable Development, Chinese Academy of Agricultural Sciences (Beijing, China). Strains B2–B4 were provided by the Biocontrol Laboratory, Institute of Plant Protection, Guangdong Academy of Agricultural Sciences (Guangzhou, China). Strain B5 was provided by the Insect Ecology Laboratory, South China Agricultural University (Guangzhou, China). Strain B6 was collected from *B. dorsalis* in a starfruit plantation (Boluo County, Guangdong Province, China; 23°23′N, 114°30′E).

*Beauveria bassiana* strains were incubated on Sabouraud dextrose agar with a 1% yeast (SDAY) medium at 25°C for 7–10 days, with a 16:8-h (light:dark) regime (Pan et al., 2008; Zhang et al., 2010). Conidia were isolated using the method of Ekesi et al. (2003), after which the mature conidia were diluted using 0.1% Tween-80 in sterile water to prepare a suspension with a concentration of 1.0 × 10^8^ conidia/mL. A solution of 0.1% Tween-80 in sterile water was used as the control.

### 2.2 Insect collection and rearing

Fallen fruits (carambola) were collected from Boluo County (Huizhou City, Guangdong Province), Conghua, Tuhua, and Popcorn Park in Guangzhou (Guangdong Province). *Bactrocera dorsalis* larvae were collected from the fruits and cultured in the laboratory. Larvae were reared on an artificial diet for two generations before testing was initiated, which enabled us to accurately identify their adults and establish pure breeding colonies of *B. dorsalis*. Larvae were reared indoors on an artificial diet (Jin et al., 2011b) and adults were fed a 1:1 yeast/sugar artificial diet and water (Liu et al., 2017). The diet described by Jin et al. (2011b) contains corn gain, banana, sodium benzoate, yeast, sucrose, winding paper, hydrochloric acid, and water. Different *B. dorsalis* life stages were cultured in the laboratory (temperature: 26 ± 1°C, relative humidity [RH]: 60–70%, and 14:10-h light:dark photoperiod) (Li et al., 2023). Two natural enemies of *B. dorsalis*, the predator *Amblyseius cucumeris* (Mesostigmata: Phytoseiidae) and the parasitoid *Anastatus japonicus* (Hymenoptera: Eupelmidae), were provided by the Biological Control Group, Institute of Plant Protection, Guangdong Academy of Agricultural Sciences. *Amblyseius cucumeris* was raised on bran and fed with *Aleuroglyphus ovatus* (Acari: Acaridae) (temperature: 26 ± 1°C, RH: 70 ± 5%, and 16:8-h light:dark photoperiod) (Zhang et al., 2002; Li, 2017). *Anastatus japonicus* adults were reared in 10% honey water, and eggs of *Antheraea pernyi* (Lepidoptera: Saturniidae) were used as alternative hosts for offspring reproduction (temperature: 24 ± 1°C, RH: 70 ± 5%, and 16:8-h light:dark photoperiod) (Zhao et al., 2023).

### 2.3 Insecticide

We tested several current pesticides routinely applied in local Chinese orchards. We purchased 40% Phoxim emulsifiable concentrate (EC) from Shandong Shengbang Lunan Pesticide Co (active ingredient: phoxim, CAS: 14816–18-3). We acquired 45% Malathion EC from Guangdong Gaozhou Chemical General Factory (active ingredient: malathion, CAS: 121–75-5). We purchased 48% Lorsban (EC) from Dow Agro, USA (active ingredient: chlorpyrifos, CAS: 2921-88-2). We procured 1.8% Avermectin EC from Zhejiang Shenghua Baike Biological Co (active ingredient: avermectin, CAS: 71751–41-2). A 10% Glyphosate aqueous solution (AS) was produced by Nantong Jiangshan Pesticide Chemical Co., Ltd. (active ingredient: glyphosate, CAS: 1071-83-6). Lastly, a 20% Paraquat AS was sourced from Hubei Shalongda Co (active ingredient: paraquat, CAS: 4685-14-7).

### 2.4 Determining *Beauveria bassiana* virulence

To accurately determine the virulence of the six strains of *B. bassiana*, the 1.0 × 10^8^ concentration was used to determine its pathogenicity to *B. dorsalis* (Pan et al., 2008). Eggs, 3rd instar larvae, and pupae of *B. dorsalis* were dipped in a spore suspension of 1.0 × 10^8^ conidia/mL for 30 s (Pan et al., 2008). The eggs were then placed in a rearing box with artificial feed. We used 3rd instar larvae because, in the field, the first two larval instars feed within the host plant; after reaching the third instar, they emerge from the host plant and drop to the soil to pupate (Susanto et al., 2022). At this point, they may come into contact with other materials and substrates, including soil containing *B. bassiana*. Therefore, only 3rd instar larvae were tested.

The 3rd instar larvae and pupae were transferred to plastic boxes containing moist sandy soil (water mixed with sandy soil, keeping the surface of the sandy soil in contact with water, but not saturating it, to avoid particles sticking together) and reared at 25°C. Egg hatching, larval pupation, and pupal emergence was recorded daily. Adults were treated using the film method (Lohse et al., 2015). Specifically, we used a hand-held sprayer to evenly spray an equal volume (5 mL) of the suspension containing 1.0 × 10^8^ conidia/mL on the internal wall of a 250-mL triangular bottle. The suspension was air dried. We collected an initial 30 emerging adults over 1 to 2 days, while adding feed and water, and observed the mortality rate over 15 days. Thirty eggs (freshly laid eggs, 1–2 h old), 3rd instar larvae, pupae (1–2 days after pupation), and adults were used in each treatment (six different *B. bassiana* strains for each *B. dorsalis* life stage), with five replicates of observations starting from the third day of the treatment (observations were made up to day 15, five times in total).

### 2.5 Strain B4 biological trait determination

After initial screening showed that the B4 strain was the most virulent of the six tested, we diluted *B. bassiana* (B4) into five concentrations, i.e., 1.0 × 10^3^, 1.0 × 10^4^, 1.0 × 10^5^, 1.0 × 10^6^, and 1.0 × 10^7^ conidia/mL. The *B. dorsalis* treatment followed the procedure described in Section 2.4. Observations were made daily after treatment, and mortality was recorded for each treatment for 20 days. We calculated the mortality, lethal mid-concentration (LC_50_), and lethal mid-time (LT_50_).

### 2.6 Effect of environmental factors on the virulence of the B4 strain

The effect of temperature on the virulence of the B4 strain was tested at eight different temperatures (13, 16, 19, 22, 25, 28, 31, and 34°C, observed for 10 days). The flies were reared at 95% relative humidity and 16:8-h light:dark cycle in an artificial climatic chamber to observe the effect of temperature on mortality. The treatment of the different *B. dorsalis* life stages with strain B4 followed the procedures outlined in Section 2.4.

The effect of RH on the virulence of the B4 strain involved exposing different *B. dorsalis* life stages to a constant relative humidity in four different ranges, i.e., 60–70, 71–80, 81–90, and 91–100% RH. The treatment of the different *B. dorsalis* life stages with strain B4 followed the procedures outlined in Section 2.4. The larvae, pupae, and adults were observed for 10 days at 25°C after inoculation.

The combined effect of temperature and RH on B4 strain growth was also assessed. Discs were collected from the edge of B4 colonies that had been cultured for 7 days using a 5-mm diameter paper punch. The discs were inoculated into the center of SDAY medium, marked with two points at the bottom of the Petri dish to highlight the original diameter, and incubated under different temperature and RH conditions. Subsequently, the diameter of the colonies was measured every 2 days. The final data was recorded on the 10th day (with five replicates).

To determine the effect of the relative soil moisture content on the virulence of the B4 strain, we used loose sandy soil with a field water holding capacity of 4.5%. Before test initiation, the soil was sieved to remove impurities and then baked in an oven at 105°C for 5–6 h until it reached a constant mass. Six different relative soil water levels (40, 50, 60, 70, 80, and 90%) were obtained following the protocol of Hou et al. (2022). We sprayed 5 mL of a 1.0 × 10^7^ conidia/mL suspension evenly on the soil surface at different RHs with a hand-held sprayer. The 3rd instar larvae were then placed on the soil and the number of larvae pupating was recorded daily (six treatments, 30 larvae per treatment, five replicates). For the pupal virulence test, pupae that had pupated 1–2 days prior were placed on soil with different relative water contents, a hand-held sprayer was used to apply 5 mL of a suspension of 1.0 × 10^7^ conidia/mL to the soil surface, and the number of emerging adults was recorded daily (six treatments, 30 pupae per treatment, five replicates). The relative water content of the test soils was maintained by weighing the samples at 12 h intervals at 25°C and supplementing them with water to account for any evaporatory loss to maintain a consistent water content.

### 2.7 Effect of common chemical agents on the biological characteristics of the B4 strain

The six insecticides were tested at three different concentrations. The first concentration involved using the recommended field concentration (concentrations recommended for routine use in the instructions), which is considered a lethal dose. The second concentration was the conventionally used concentration diluted five times, considered a sublethal dose, whereas the third concentration was the conventionally used concentration diluted ten times, considered a low sublethal dose (Xu et al., 2002).

The B4 strain, cultured for 10 days (25°C, 16:8-h light:dark), was used to prepare a conidial suspension using sterile water containing 0.1% Tween-80. Each agent was adjusted to the desired concentration using 1 mL of B4 conidia suspension containing 5% sucrose. We transferred 100 μL of the solution to a sterile slide and placed it in a Petri dish lined with filter paper, added sterile water dropwise to the dish to maintain 100% RH, and incubated it for 24 h to observe spore germination using a microscope (Sigma-Aldrich Chemie GmbH, Taufkirchen, Germany; in five replications).

We applied 0.1 mL of the insecticide solution to evenly coat the surface of the SDAY medium. We transferred a 5-mm diameter disc of the B4 strain (cultured at 25°C and 95% RH for 7 days) to the center of the medium containing the insecticide. The sampling method of the disc followed the procedure described in Section 2.6. We marked two points at the bottom of the Petri dish to highlight the initial diameter; each treatment was replicated five times. The colony diameter was measured on the 5th and 10th d. We used the culture medium without pesticide as the control.

### 2.8 Effect of strain B4 on the natural enemies of *Bactrocera dorsalis*

The effect of strain B4 on *A. cucumeris* was assessed. The concentration of spore powder in 0.1 g was calculated to be 7.0 × 10^9^ conidia/g using haemocytometry (Mulatu et al., 2021). We created three treatments of *B. bassiana* with 0.5, 2.5, and 12.5 g (based on pre-experimental results and the characteristics of the feeding patterns of *A. cucumeris*) (Zhang et al., 2002; Li, 2017). We mixed the samples with 500 g of bran and 10 g of seeds containing approximately 300 mites (in five replicates). The mite population was observed on the 10th and 20th days after inoculation. Five samples (of 0.1 g each) were randomly collected and observed using a dissecting microscope.

The effect of strain B4 on *A. japonicus* involved different concentrations (based on the results of the previous experiments and the characteristics of the feeding patterns of *A. japonicus*) (Zhao et al., 2023). Spore suspensions of 1.0 × 10^5^, 1.0 × 10^6^, 1.0 × 10^7^, 1.0 × 10^8^, and 1.0 × 10^9^ conidia/mL were evenly sprayed on the wall of a tube (sterilized glass tubes with a diameter of 15 cm and a height of 20 cm) and air dried. Each tube was inoculated with 50 *A. japonicus* at 1–2 days after emergence and 50 *A. pernyi* eggs. Parasitic holes in the eggs were evaluated every 5 days, and the experiment was terminated on day 20. Parasitized eggs were recorded for each treatment.

### 2.9 Statistical analysis

Calculations of LC_50_ and LT_50_ with corresponding 95% confidence limits (CL) were first corrected for mortality and then subjected to probit regression analyses using the SPSS v.22.0 software (SPSS Inc., Chicago, IL). The SAS software (version 9.4) was used for the other data analyses. The experimental results were analyzed using Duncan’s multiple range test (DMRT), with *p* < 0.05 considered statistically significant. Normal distribution was verified before data analysis, followed by ANOVA. The calculation of the corrected mortality was as follows:


$$\begin{array}{l} \mathrm{Correction of mortality}\;\left(\%\right)=\\ \frac{\mathrm{treatment mortality}-\mathrm{control mortality}}{1-\mathrm{control mortality}}\times 100 \end{array}$$

The calculation of the growth inhibition rate of *B. bassiana* colonies was as follows:


$$\begin{array}{l} \mathrm{Inhibition rate}\;\left(\%\right)=\\ \frac{\begin{array}{l} \mathrm{control}\;\mathrm{Petri}\;\mathrm{dish colony diameter}-\\ \mathrm{dosing}\;\mathrm{Petri}\;\mathrm{dish colony diameter} \end{array}}{\mathrm{control}\;\mathrm{Petri}\;\mathrm{dish colony diameter}}\times 100 \end{array}$$

## 3 Results

### 3.1 Virulence of different strains of *Beauveria bassiana* against *Bactrocera dorsalis*

The six strains of *B. bassiana* were not highly virulent to *B. dorsalis* eggs (Table 1). For 3rd instar larvae, pupae, and adults, the virulence of each strain increased with increasing treatment duration. Strain B4 had the highest virulence in every observation, reaching 52.67% (3rd instar larvae), 61.33% (pupae), and 90.67% (adults) at day 15, which was significantly higher than that of all the other strains (Table 1). Therefore, we focused entirely on strain B4 in all subsequent experiments.

**Table 1:** Virulence of different *B. bassiana* strains against *B. dorsalis* at the concentration of 1.0 × 10^8^ conidia/mL.

Stage	No.	Average mortality (%)				
		3 d	5 d	7 d	10 d	15 d
Egg	B1	4.67 ± 0.06 ^a^	4.67 ± 0.06 ^a^	3.33 ± 0.10 ^a^	3.33 ± 0.10 ^a^	3.33 ± 0.10 ^a^
	B2	0 ^b^	0 ^b^	0 ^b^	0 ^b^	0 ^b^
	B3	10.00 ± 0.32 ^c^	2.00 ± 0.35 ^c^	0 ^b^	0 ^b^	0 ^b^
	B4	1.34 ± 0.01 ^d^	1.33 ± 0.01 ^d^	1.33 ± 0.01 ^c^	1.33 ± 0.01 ^c^	1.33 ± 0.01 ^c^
	B5	0.67 ± 0.04 ^e^	0.67 ± 0.04 ^e^	0.67 ± 0.04 ^d^	0.67 ± 0.04 ^d^	0.67 ± 0.04 ^d^
	B6	0 ^b^	0 ^b^	0 ^b^	0 ^b^	0 ^b^
	Control	0 ^b^	0 ^b^	0 ^b^	0 ^b^	0 ^b^
3rd instar larvae	B1	3.33 ± 0.64 ^b^	4.67 ± 0.65 ^b^	8.67 ± 0.61 ^b^	20.67 ± 0.64 ^b^	36.67 ± 1.44 ^c^
	B2	1.33 ± 0.02 ^cd^	2.00 ± 0.32 ^cd^	4.00 ± 0.59 ^c^	14.00 ± 1.29 ^d^	26.00 ± 1.82 ^d^
	B3	0.67 ± 0.01 ^d^	2.67 ± 0.34 ^c^	5.33 ± 0.35 ^c^	16.00 ± 1.04 ^cd^	28.00 ± 0.64 ^d^
	B4	5.33 ± 0.64 ^a^	6.67 ± 0.88 ^a^	12.67 ± 0.91 ^a^	28.67 ± 1.29 ^a^	52.67 ± 1.29 ^a^
	B5	4.00 ± 0.22 ^b^	5.33 ± 0.33 ^ab^	11.33 ± 0.59 ^a^	24.67 ± 1.44 ^a^	40.67 ± 1.04 ^b^
	B6	2.00 ± 0.25 ^c^	2.67 ± 0.22 ^c^	7.33 ± 0.42 ^b^	19.33 ± 1.04 ^bc^	33.33 ± 0.64 ^c^
	Control	0 ^e^	0.67 ± 0.05 ^d^	2.00 ± 0.22 ^d^	2.00 ± 0.18 ^e^	3.33 ± 0.64 ^e^
Pupae	B1	4.00 ± 0.57 ^b^	5.33 ± 0.58 ^c^	11.33 ± 1.04 ^b^	24.67 ± 1.84 ^bc^	45.33 ± 2.25 ^b^
	B2	2.00 ± 0.19 ^c^	2.67 ± 0.34 ^cd^	8.67 ± 0.91 ^bc^	21.33 ± 1.55 ^bc^	34.00 ± 2.07 ^c^
	B3	0.67 ± 0.08 ^d^	2.00 ± 0.27 ^d^	6.67 ± 0.60 ^c^	18.67 ± 1.19 ^c^	23.88 ± 1.64 ^d^
	B4	6.00 ± 0.29 ^a^	9.33 ± 1.04 ^a^	15.33 ± 1.84 ^a^	34.67 ± 2.25 ^a^	61.33 ± 3.54 ^a^
	B5	4.00 ± 0.35 ^ab^	7.33 ± 0.66 ^b^	12.00 ± 1.23 ^ab^	27.33 ± 2.47 ^b^	47.33 ± 1.93 ^b^
	B6	2.67 ± 0.32 ^c^	4.00 ± 0.86 ^cd^	9.33 ± 1.55 ^bc^	23.33 ± 1.68 ^bc^	40.00 ± 2.47 ^bc^
	Control	0 ^e^	0 ^e^	1.33 ± 0.09 ^d^	1.33 ± 0.10 ^d^	2.00 ± 0.18 ^e^
Adult	B1	18.00 ± 1.44 ^b^	40.00 ± 3.00 ^ab^	58.00 ± 2.85 ^c^	70.67 ± 3.10 ^ab^	78.67 ± 1.04 ^b^
	B2	13.33 ± 1.04 ^cd^	28.00 ± 3.15 ^c^	42.67 ± 1.68 ^d^	65.33 ± 2.16 ^b^	71.33 ± 1.44 ^cd^
	B3	14.00 ± 1.41 ^c^	22.00 ± 1.03 ^c^	39.33 ± 2.32 ^d^	60.00 ± 6.25 ^b^	67.33 ± 1.82 ^d^
	B4	22.67 ± 1.03 ^a^	46.67 ± 1.68 ^a^	75.33 ± 2.33 ^a^	80.67 ± 2.65 ^a^	90.67 ± 1.47 ^a^
	B5	20.0 ± 1.01 ^ab^	44.00 ± 0.91 ^ab^	66.00 ± 2.25 ^b^	73.33 ± 1.68 ^ab^	80.67 ± 2.73 ^b^
	B6	10.0 ± 0.65 ^d^	38.67 ± 2.71 ^b^	54.00 ± 1.29 ^c^	68.00 ± 3.06 ^b^	76.00 ± 2.89 ^bc^
	Control	0 ^e^	1.33 ± 0.15 ^d^	3.33 ± 0.64 ^e^	4.00 ± 0.19 ^c^	5.33 ± 0.14 ^e^

Data with the same number and letter in the same column indicate no significant difference in the Duncan’s Multiple Range Test (DMRT) test (*p* ≥ 0.05).

### 3.2 Biological traits of strain B4

The corrected mortality rates for *B. dorsalis* were 1.33% (eggs), 51.04% (3rd instar larvae), 60.54% (pupae), and 90.14% (adults). The virulence of strain B4 on the different life stages of *B. dorsalis* decreased from adults > pupae >3rd larvae > eggs. Strain B4 did not affect eggs. The LC_50_ of the B4 strain on *B. dorsalis* was 0.79 × 10^7^ conidia/mL on larvae (95% CL of 2.59 × 10^6^–2.40 × 10^7^), 0.78 × 10^6^ conidia/mL on pupae (95% CL of 3.63 × 10^5^–1.67 × 10^6^), and 1.70 × 10^5^ conidia/mL on adults (95% CL of 1.08 × 10^5^–2.68 × 10^5^).

At the same concentration (except for 1.0 × 10^3^ conidia/mL), there were some differences in the LT_50_ between the larvae, pupae, and adults, suggesting that tolerance to B4 varies between the three life stages. The LT_50_ of each *B. dorsalis* stage showed a decreasing trend with increasing B4 strain concentration (Table 2). Strain B4 was most toxic to adults, and the LT_50_ was less than 10 days at concentrations as low as 1.0 × 10^6^ conidia/mL.

**Table 2:** LT_50_ of the B4 strain at different concentrations in *B. dorsalis.*

Stage	LT_50_ (day)				
	1.0 × 10^3^ conidia/mL	1.0 × 10^4^ conidia/mL	1.0 × 10^5^ conidia/mL	1.0 × 10^6^ conidia/mL	1.0 × 10^7^ conidia/mL
Larvae	25.9 ± 1.73 ^a^	23.7 ± 0.98 ^a^	20.5 ± 2.31 ^a^	14.2 ± 1.15 ^a^	13.9 ± 0.88 ^a^
Pupae	25.5 ± 1.17 ^a^	22.1 ± 0.58 ^ab^	19.7 ± 1.15 ^a^	13.4 ± 0.35 ^a^	12.5 ± 0.87 ^a^
Adult	22.9 ± 0.87 ^a^	20.1 ± 0.58 ^b^	11.2 ± 1.73 ^b^	7.1 ± 1.10 ^b^	6.3 ± 0.40 ^b^

Data with the same number and letter in the same column are not significantly different using the DMRT test (*p* ≥ 0.05).

### 3.3 Environmental effects on the virulence of the B4 strain

The mortality rate of *B. dorsalis* increased with temperature (from 13–25°C), peaking around 25°C and gradually decreasing from 25–34°C (except for the 3rd instar larvae at 0.56 × 10^11^ conidia/mL). The toxic effect of strain B4 on 3rd instar larvae, pupae, and adults peaked at 25°C, with mortality reaching 52.00, 60.00, and 86.67% at a concentration of 1.0 × 10^7^ conidia/mL, respectively. At 25°C, the LC_50_ of strain B4 was the lowest at concentrations of 0.81 × 10^7^ (larvae), 0.09 × 10^7^ (pupae), and 0.02 × 10^7^ conidia/mL (adults) (Table 3). Similarly, the B4 strain had the highest growth and fastest growth rate at 25°C, followed by 28°C (Table 4).

**Table 3:** Cumulative mortality and toxicity of the B4 strain against *B. dorsalis* in different temperatures.

Stage	Temp (°C)	Cumulative mortality (%)					LC_50_ (95%CL) (conidia/mL)	R
		1.0 × 10^3^ conidia/mL	0.56 × 10^11^ conidia/mL	1.0 × 10^5^ conidia/mL	1.0 × 10^6^ conidia/mL	1.0 × 10^7^ conidia/mL		
3^rd^ instar larvae	13	0.67 ± 0.07 ^e^	1.15 × 10^11^	5.33 ± 1.09 ^f^	8.67 ± 1.08 ^e^	10.67 ± 1.05 ^f^	0.56 × 10^11^ (3. 63 × 10^8^–0.87 × 10^13^)	0.9513*
	16	1.33 ± 0.05 ^de^	8.71 × 10^7^	7.33 ± 1.32 ^f^	10.00 ± 1.01 ^e^	12.67 ± 1.18 ^f^	1.15 × 10^11^ (3.95 × 10^8^–3.37 × 10^13^)	0.9550*
	19	5.33 ± 0.09 ^bc^	4.43 × 10^7^	16.00 ± 1.18 ^cd^	24.67 ± 1.91 ^cd^	39.33 ± 1.45 ^de^	8.71 × 10^7^ (1.52 × 10^7^–49.88 × 10^7^)	0.9980*
	22	6.00 ± 0.68 ^b^	0.81 × 10^7^	19.33 ± 0.60 ^bc^	27.33 ± 2.67 ^bc^	42.67 ± 0.44 ^cd^	4.43 × 10^7^ (0.92 × 10^7^–21.37 × 10^7^)	0.9975*
	25	8.67 ± 0.45 ^a^	1.47 × 10^7^	28.67 ± 0.49 ^a^	34.67 ± 1.36 ^a^	52.00 ± 0.59 ^a^	0.81 × 10^7^ (0.26 × 10^7^–2.52 × 10^7^)	0.9926*
	28	7.33 ± 1.36 ^ab^	2.14 × 10^7^	23.33 ± 0.99 ^b^	33.33 ± 1.50 ^a^	48.00 ± 1.23 ^ab^	1.47 × 10^7^ (0.42 × 10^7^–5.15 × 10^7^)	0.9994*
	31	6.00 ± 0.50 ^b^	11.45 × 10^7^	20.67 ± 1.36 ^b^	31.33 ± 0.45 ^ab^	45.33 ± 1.95 ^bc^	2.14 × 10^7^ (0.57 × 10^7^–8.00 × 10^7^)	0.9998*
	34	3.33 ± 0.03 ^cd^	6.67 ± 0.08 ^d^	14.00 ± 1.00 ^de^	22.67 ± 0.53 ^cd^	34.67 ± 1.04 ^e^	11.45 × 10^7^ (2.03 × 10^7^–64.57 × 10^7^)	0.9993*
Pupae	13	0.67 ± 0.05 ^f^	6.00 ± 0.59 ^f^	9.33 ± 0.73 ^e^	12.00 ± 1.54 ^f^	14.67 ± 0.75 ^e^	4.47 × 10^9^ (1.43 × 10^8^–1.39 × 10^11^)	0.8964*
	16	2.67 ± 0.06 ^e^	6.67 ± 0.13 ^f^	11.33 ± 1.95 ^e^	12.67 ± 2.09 ^f^	16.00 ± 1.61 ^e^	1.12 × 10^11^ (3.15 × 10^8^–4.01 × 10^13^)	0.9515*
	19	7.33 ± 0.45 ^c^	20.00 ± 0.90 ^cd^	31.33 ± 1.03 ^cd^	38.00 ± 2.45 ^cd^	43.33 ± 0.43 ^d^	0.76 × 10^7^ (0.24 × 10^7^–2.43 × 10^7^)	0.9711*
	22	8.00 ± 0.59 ^c^	22.00 ± 1.36 ^c^	34.00 ± 0.95 ^bc^	42.67 ± 0.45 ^bc^	52.67 ± 1.18 ^c^	0.34 × 10^7^ (0.13 × 10^7^–0.90 × 10^7^)	0.97448
	25	12.67 ± 0.94 ^a^	33.33 ± 1.44 ^a^	42.67 ± 1.21 ^a^	50.00 ± 0.86 ^a^	60.00 ± 1.23 ^a^	0.09 × 10^7^ (0.04 × 10^7^–0.20 × 10^7^)	0.9559*
	28	10.67 ± 0.41 ^b^	30.67 ± 1.12 ^ab^	40.00 ± 2.27 ^a^	47.33 ± 1.04 ^ab^	57.33 ± 1.04 ^ab^	0.14 × 10^7^ (0.06 × 10^7^–0.33 × 10^7^)	0.9532*
	31	10.00 ± 0.68 ^b^	27.33 ± 1.05 ^b^	38.67 ± 0.73 ^ab^	46.67 ± 2.01 ^ab^	54.00 ± 1.14 ^bc^	0.21 × 10^7^ (0.08 × 10^7^–0.53 × 10^7^)	0.9571*
	34	4.67 ± 0.08 ^d^	17.33 ± 1.06 ^de^	27.33 ± 0.87 ^d^	34.00 ± 1.82 ^de^	40.00 ± 0.23 ^d^	2.00 × 10^7^ (0.50 × 10^7^–7.98 × 10^7^)	0.9454*
Adult	13	1.33 ± 0.03 ^e^	6.67 ± 0.01 ^f^	10.00 ± 1.36 ^f^	12.67 ± 0.58 ^f^	16.00 ± 1.05 ^f^	1.15 × 10^10^ (1.90 × 10^8^–0.69 × 10^12^)	0.9238*
	16	2.67 ± 0.08 ^e^	7.33 ± 0.15 ^f^	11.33 ± 0.59 ^f^	14.67 ± 0.77 ^f^	16.67 ± 1.38 ^f^	4.54 × 10^10^ (2.47 × 10^8^–0.83 × 10^13^)	0.9501*
	19	7.33 ± 0.07 ^cd^	13.33 ± 0.74 ^de^	21.33 ± 0.90 ^e^	40.67 ± 1.09 ^de^	65.33 ± 0.74 ^d^	0.25 × 10^7^ (0.12 × 10^7^–0.53 × 10^7^)	0.9879*
	22	9.33 ± 0.45 ^c^	16.00 ± 1.81 ^cd^	26.67 ± 0.77 ^d^	45.33 ± 2.27 ^cd^	72.67 ± 0.81 ^c^	0.11 × 10^7^ (0.06 × 10^7^–0.20 × 10^7^)	0.9861*
	25	18.00 ± 0.82 ^a^	26.00 ± 0.95 ^a^	44.00 ± 0.83 ^a^	54.67 ± 1.87 ^a^	86.67 ± 1.21 ^a^	0.02 × 10^7^ (0.01 × 10^7^–0.03 × 10^7^)	0.9673*
	28	14.00 ± 1.22 ^b^	21.33 ± 0.58 ^b^	35.33 ± 1.14 ^b^	51.33 ± 1.51 ^ab^	82.00 ± 0.95 ^b^	0.03 × 10^7^ (0.02 × 10^7^–0.06 × 10^7^)	0.9757*
	31	12.67 ± 0.03 ^b^	18.00 ± 0.73 ^bc^	31.33 ± 0.92 ^c^	49.33 ± 0.60 ^bc^	76.67 ± 1.85 ^c^	0.06 × 10^7^ (0.03 × 10^7^–0.11 × 10^7^)	0.9807*
	34	6.00 ± 0.04 ^d^	10.00 ± 0.86 ^ef^	20.00 ± 0.68 ^e^	36.00 ± 1.25 ^ef^	60.67 ± 0.83 ^e^	0.44 × 10^7^ (0.20 × 10^7^–1.01 × 10^7^)	0.9903*

CL, confidence limits; R, correlation index; Data with the same number and letter in the same column have *P* ≥ 0.05 (DMRT test); *Significant difference (*P* ≤ 0.05).

**Table 4:** Effect of temperature on the growth of the B4 strain.

Temp (°C)	Colony diameter (cm)				
	2 d	4 d	6 d	8 d	10 d
13	0.50 ± 0.02 ^e^	0.90 ± 0.08 ^d^	1.20 ± 0.08 ^e^	1.90 ± 0.28 ^e^	2.50 ± 0.18 ^e^
16	0.70 ± 0.03 ^de^	1.00 ± 0.08 ^cd^	2.00 ± 0.21 ^d^	3.10 ± 0.21 ^d^	3.90 ± 0.22 ^d^
19	0.90 ± 0.09 ^cd^	1.40 ± 0.10 ^bc^	2.50 ± 0.17 ^cd^	4.00 ± 0.22 ^bc^	5.20 ± 0.24 ^bc^
22	1.10 ± 0.11 ^bc^	1.70 ± 0.08 ^b^	2.70 ± 0.18 ^bc^	4.50 ± 0.20 ^b^	5.80 ± 0.22 ^b^
25	1.50 ± 0.14 ^a^	2.40 ± 0.21 ^a^	3.90 ± 0.16 ^a^	6.90 ± 0.24 ^a^	7.70 ± 0.27 ^a^
28	1.40 ± 0.12 ^a^	2.20 ± 0.11 ^a^	3.50 ± 0.21 ^a^	6.40 ± 0.14 ^a^	7.00 ± 0.22 ^a^
31	1.40 ± 0.08 ^ab^	2.10 ± 0.17 ^a^	3.20 ± 0.19 ^ab^	6.30 ± 0.13 ^a^	6.80 ± 0.16 ^a^
34	0.80 ± 0.04 ^de^	1.20 ± 0.09 ^cd^	2.10 ± 0.18 ^d^	3.80 ± 0.17 ^c^	4.90 ± 0.24 ^c^

Data with the same number and letter in the same column indicate no significant difference in the DMRT test (*p* ≥ 0.05).

A low RH reduced the mortality of *B. dorsalis* caused by the B4 strain. The highest mortality rates were achieved at 91–100% RH (1.0 × 10^7^ conidia/mL), and were 89.33% (adults), 63.33% (pupae), and 52.67% (larvae). Likewise, the minimum LC_50_ occurred at 91–100% RH, with 1.57 × 10^5^ conidia/mL (adults), 0.72 × 10^6^ conidia/mL (pupae), and 0.60 × 10^6^ (larvae) conidia/mL (Table 5). The *B. bassiana* growth was significantly higher at 91–100 and 81–90% RH than in all of the other treatments. An RH of 91–100% is the optimum growth humidity for B4 (Table 6).

**Table 5:** Cumulative mortality and toxicity of the B4 strain against *B. dorsalis* with various humidity.

Stage	RH (%)	Cumulative mortality (%)					LC_50_ (95%CL) (conidia/mL)	*R*
		1.0 × 10^3^ conidia/mL	0.56 × 10^11^ conidia/mL	1.0 × 10^5^ conidia/mL	1.0 × 10^6^ conidia/mL	1.0 × 10^7^ conidia/mL		
3^rd^ instar larvae	91–100	7.33 ± 0.05 ^a^	18.00 ± 1.19^a^	25.33 ± 1.20 ^a^	38.67 ± 2.03 ^a^	52.67 ± 0.88 ^a^	0.60 × 10^6^ (0.21 × 10^7^–1.67 × 10^7^)	0.9943*
	81–90	5.33 ± 0.10 ^b^	14.00 ± 1.14^b^	20.00 ± 0.49 ^b^	31.33 ± 1.29 ^b^	46.00 ± 2.07 ^b^	1.89 × 10^7^ (0.52 × 107–6.81 × 10^7^)	0.9937*
	71–80	2.00 ± 0.06 ^c^	8.00 ± 0.48^c^	14.00 ± 0.98 ^c^	23.33 ± 0.69 ^c^	34.00 ± 1.07 ^c^	0.73 × 10^8^ (1.58 × 10^7^–33.78 × 10^7^)	0.9872*
	61–70	1.33 ± 0.02 ^d^	6.00 ± 0.35 ^c^	11.33 ± 0.41 ^d^	18.00 ± 0.74 ^d^	26.00 ± 1.65 ^d^	2.76 × 10^8^ (3.78 × 10^7^–200.85 × 10^7^)	0.9794*
Pupae	91–100	12.67 ± 0.29 ^a^	28.67 ± 2.46 ^a^	41.33 ± 1.61 ^a^	52.00 ± 0.98 ^a^	63.33 ± 1.33 ^a^	0.72 × 10^6^ (0.04 × 10^7^–0.14 × 10^7^)	0.9858*
	81–90	8.67 ± 0.05 ^b^	24.00 ± 0.96 ^a^	32.67 ± 0.82 ^b^	44.00 ± 1.30 ^b^	56.00 ± 1.93 ^b^	2.55 × 10^6^ (0.10 × 10^7^–0.63 × 10^7^)	0.9806*
	71–80	5.33 ± 0.07 ^c^	15.33 ± 1.47 ^b^	24.67 ± 1.44 ^c^	33.33 ± 1.04 ^c^	44.67 ± 1.19 ^c^	1.54 × 10^7^ (0.43 × 10^7^–5.44 × 10^7^)	0.9839*
	60–70	4.67 ± 0.16 ^d^	10.00 ± 1.40 ^b^	14.00 ± 0.30 ^d^	26.00 ± 1.12 ^d^	34.00 ± 0.69 ^d^	1.64 × 10^8^ (2.21 × 10^7^–120.90 × 10^7^)	0.9943*
Adult	91–100	16.67 ± 0.35 ^a^	24.00 ± 0.54 ^a^	40.67 ± 0.64 ^a^	56.00 ± 1.75 ^a^	89.33 ± 2.47 ^a^	1.57 × 10^5^ (0.01 × 10^7^–0.02 × 10^7^)	0.9628*
	81–90	13.33 ± 0.46 ^b^	19.33 ± 0.35 ^b^	34.67 ± 0.35 ^b^	50.67 ± 1.16 ^b^	80.67 ± 2.59 ^b^	4.14 × 10^5^ (0.02 × 10^7^–0.07 × 10^7^)	0.9779*
	71–80	8.67 ± 0.19 ^c^	14.00 ± 0.52 ^c^	26.67 ± 1.44 ^c^	40.67 ± 1.61 ^c^	73.33 ± 1.06 ^c^	1.30 × 10^6^ (0.07 × 10^7^–0.24 × 10^7^)	0.9788*
	60–70	5.33 ± 0.10 ^d^	7.33 ± 0.64 ^d^	20.67 ± 1.79 ^d^	35.33 ± 1.53 ^d^	65.33 ± 1.75 ^d^	3.31 × 10^6^ (0.16 × 10^7^–0.68 × 10^7^)	0.9819*

CL, confidence limits; R, correlation index; Data with the same number and letter in the same column indicate no significant variation in the DMRT test (*p* ≥ 0.05); *Significantly different (*P* ≤ 0.05).

**Table 6:** Effect of humidity on the growth of the B4 strain.

RH (%)	Colony diameter (cm)				
	2 d	4 d	6 d	8 d	10 d
91–100	2.00 ± 0.14 ^a^	2.80 ± 0.22 ^a^	3.70 ± 0.22 ^a^	4.20 ± 0.34 ^a^	4.90 ± 0.24 ^a^
81–90	1.90 ± 0.21 ^a^	2.30 ± 0.14 ^b^	2.90 ± 0.23 ^b^	3.40 ± 0.30 ^a^	3.90 ± 0.20 ^b^
71–80	1.40 ± 0.16 ^b^	1.60 ± 0.13 ^c^	1.90 ± 0.14 ^c^	1.90 ± 0.07 ^b^	2.10 ± 0.08 ^c^
60–70	1.10 ± 0.03 ^b^	1.20 ± 0.05 ^c^	1.20 ± 0.05 ^d^	1.40 ± 0.06 ^b^	1.50 ± 0.05 ^d^

Data with the same number and letter in the same column indicate no significant variation between them in the DMRT test (*p* ≥ 0.05).

The mortality of *B. dorsalis* between the three relative soil water contents of 50, 60, and 70% did not vary significantly. The variations in the virulence of the B4 strain between the different water contents (40, 80, and 90%) were highly significant. In addition, the highest mortality rate was 79.21% for larvae at a relative soil moisture content of 60 and 80.63% for pupae at a relative soil moisture content of 50% (Table 7).

**Table 7:** Effect of the relative soil water content on the virulence of the B4 strain.

Soil relative water content (%)	Adjusted mortality (%)	
	3rd instar larvae	Pupae
90	39.08 ± 1.50 ^c^	67.36 ± 1.90 ^b^
80	59.49 ± 2.34 ^b^	67.51 ± 1.82 ^b^
70	74.09 ± 2.02 ^a^	77.05 ± 1.23 ^a^
60	79.21 ± 1.26 ^a^	77.03 ± 1.38 ^a^
50	78.51 ± 2.70 ^a^	80.63 ± 1.68 ^a^
40	38.04 ± 2.63 ^c^	64.42 ± 1.54 ^b^

Data with the same number and letter in the same column indicate no significant variation between them by the DMRT test (*p* ≥ 0.05).

### 3.4 Common insecticide effects on the biological characteristics of the B4 strain

At three different doses, 1.8% avermectin EC had the strongest inhibitory effect on the germination of B4 conidia, and conidia were unable to germinate at the lethal dose (Table 8). The remaining five agents showed weak inhibition of conidial germination at both lethal and sublethal doses, but were not significantly different from the control at the low sublethal dose (Table 8).

**Table 8:** Effects of different dilution ratios of chemical reagents on the conidial germination rate of the B4 strain.

Chemical agent	Conidial germination rate (%)		
	Lethal dose	Sublethal dose	Low sublethal dose
40% Phoxim EC	88.45 ± 2.21 ^bc^	95.76 ± 1.22 ^b^	96.30 ± 2.35 ^ab^
45% Malathion EC	90.12 ± 3.15 ^b^	95.82 ± 0.98 ^b^	96.56 ± 0.87 ^ab^
48% Lorsban EC	87.21 ± 1.05 ^cd^	94.53 ± 0.49 ^b^	95.10 ± 1.15 ^ab^
1.8% Avermectin EC	0 ^e^	9.21 ± 1.01 ^d^	17.81 ± 0.55 ^c^
10% Glyphosate AS	85.92 ± 3.10 ^d^	91.46 ± 0.61 ^c^	94.87 ± 2.11 ^b^
20% Paraquat AS	86.15 ± 1.78 ^d^	92.61 ± 1.25 ^c^	95.13 ± 3.01 ^ab^
Control	98.45 ± 0.43 ^a^	98.02 ± 0.33 ^a^	98.34 ± 0.51 ^a^

Data with the same number and letter in the same column indicate no significant variation between them by the DMRT test (*p* ≥ 0.05).

Similarly, 1.8% avermectin EC had the strongest inhibitory effect on the growth of B4 strains at lethal and sublethal concentrations, followed by 48% Lorsban EC (Table 9). While 1.8% abamectin EC still had a strong inhibitory effect on B4 strain growth at sublethal doses, there was no difference between the other five agents. Therefore, the effect of 1.8% abamectin EC on the growth of the B4 strain at low sublethal concentrations was further measured. It was found that the inhibitory effect was still strong (57.14% on day 5 and 71.05% on day 10). This indicates that avermectin cannot be used in conjunction with B4.

**Table 9:** Growth inhibition rate of the B4 strain using various chemical agent doses.

Dose	Chemical agent	Dilution (times)	Inhibition rate (%)	
			5 d	10 d
Lethal dose	40% Phoxim EC	1,500	1.07 ± 0.01 ^c^	1.32 ± 0.03 ^c^
	45% Malathion EC	1,500	0.71 ± 0.02 ^c^	1.97 ± 0.01 ^c^
	48% Lorsban EC	1,000	8.21 ± 0.05 ^b^	11.05 ± 0.71 ^b^
	1.8% Avermectin EC	3,000	75.20 ± 2.25 ^a^	86.85 ± 2.01 ^a^
	10% Glyphosate AS	300	0.71 ± 0.02 ^c^	4.34 ± 0.22 ^c^
	20% Paraquat AS	250	0.71 ± 0.02 ^c^	4.21 ± 0.21 ^c^
Sublethal dose	40% Phoxim EC	7,500	0.36 ± 0.05 ^b^	0.13 ± 0.03 ^b^
	45% Malathion EC	7,500	0.36 ± 0.04 ^b^	0.26 ± 0.03 ^b^
	48% Lorsban EC	5,000	0.71 ± 0.07 ^b^	0.53 ± 0.04 ^b^
	1.8% Avermectin EC	15,000	71.43 ± 2.90 ^a^	81.58 ± 2.82 ^a^
	10% Glyphosate AS	1,500	0.36 ± 0.03 ^b^	0.53 ± 0.06 ^b^
	20% Paraquat AS	750	0.36 ± 0.05 ^b^	0.53 ± 0.12 ^b^

Data with the same number and letter in the same column indicate no significant variation between them in the DMRT test (*p* ≥ 0.05).

### 3.5 Effect of the B4 strain on natural enemies of *Bactrocera dorsalis*

The B4 strain significantly inhibited *A. cucumeris* reproduction, and the inhibitory effect increased with increasing treatment concentrations. Extending the treatment time weakened the inhibitory effect of the B4 strain on the mites (Table 10). For *A. cucumeris*, increasing both the treatment time and concentration significantly increased the mortality, especially at high concentrations of 1.0 × 10^9^ conidia/mL. More than half the mites died by day 15 (52%) (Table 11).

**Table 10:** Lethal effect of the B4 strain on *A. cucumeris.*

Density (g)	Number of predatory mites	
	10 d	20 d
0.5	33.13 ± 1.39 ^b^	56.42 ± 2.11 ^b^
2.5	29.72 ± 2.13 ^b^	49.12 ± 0.43 ^c^
12.5	28.96 ± 0.49 ^b^	38.23 ± 1.11 ^d^
Control	62.99 ± 3.90 ^a^	114.28 ± 3.16 ^a^

Data with the same number and letter in the same column indicate no significant variation between them in the DMRT test (*P* ≥ 0.05).

**Table 11:** Effect of the B4 strain on the cumulative mortality and fecundity of *A. japonicus.*

Test	Concentration (conidia/mL)	Processing days (d)			
		5	10	15	20
Cumulative mortality (%)	1.0 × 10^5^	2.15 ± 0.20 ^b^	2.78 ± 0.18 ^b^	4.20 ± 0.11 ^c^	4.74 ± 0.51 ^c^
	1.0 × 10^6^	3.05 ± 0.08 ^b^	3.62 ± 0.04 ^b^	5.22 ± 0.30 ^c^	8.64 ± 0.36 ^bc^
	1.0 × 10^7^	3.20 ± 0.06 ^b^	4.40 ± 0.16 ^b^	5.70 ± 0.65 ^bc^	8.86 ± 0.24 ^bc^
	1.0 × 10^8^	4.74 ± 0.23 ^b^	5.22 ± 031 ^b^	10.74 ± 0.62 ^b^	18.03 ± 0.42 ^b^
	1.0 × 10^9^	27.63 ± 1.13 ^a^	28.53 ± 1.42 ^a^	52.00 ± 1.80 ^a^	61.07 ± 2.71 ^a^
	Control	2.58 ± 0.03 ^b^	2.91 ± 0.05 ^b^	3. 42 ± 0.27 ^c^	3. 56 ± 0.12 ^c^
Fecundity (eggs/female)	1.0 × 10^5^	13.33 ± 0.36 ^ab^	19.67 ± 0.38 ^a^	28.00 ± 0.51 ^ab^	80.00 ± 1.21 ^ab^
	1.0 × 10^6^	12.00 ± 0.35 ^abc^	17.67 ± 0.21 ^ab^	26.67 ± 0.15 ^ab^	78.33 ± 0.44 ^ab^
	1.0 × 10^7^	11.67 ± 0.15 ^abc^	16.33 ± 1.13 ^b^	25.33 ± 0.42 ^ab^	70.00 ± 2.45 ^b^
	1.0 × 10^8^	11.33 ± 0.33 ^bc^	14.00 ± 0.62 ^b^	25.00 ± 1.02 ^ab^	69.33 ± 3.58 ^b^
	1.0 × 10^9^	8.67 ± 0.13 ^c^	13.00 ± 0.22 ^b^	24.33 ± 1.23 ^b^	38.00 ± 1.21 ^c^
	Control	15.33 ± 0.13 ^a^	20.67 ± 1.32 ^a^	29.00 ± 2.13 ^a^	85.00 ± 3.22 ^a^

Data with the same number and letter in the same column indicate no significant variation between them in the DMRT test (*p* ≥ 0.05).

The *A. japonicus* oviposition in the control was higher than that in the B4-treated samples at the same time, while higher treatment concentrations resulted in greater variation in *A. japonicus* than in the control (Table 11).

## 4 Discussion

Currently, in the field of biological control, EPF are used to control insect pests and have the potential to free producers from a heavy dependence on chemical pesticides (Legaspi et al., 2000; Lovett and Leger, 2017). Based on *B. bassiana*, as one of the most effective EPFs, many commercial products for biological control have been developed (Zimmermann, 2007). In the USSR, *B. bassiana* products were mainly used to control *Leptinotarsa decemlineata* (Coleoptera: Chrysomelidae) and Cydia pomonella (Lepidoptera: Tortricidae) (Ferron, 1981). In China, *B. bassiana* is widely used to control *Ostrinia nubilalis* (Lepidoptera: Crambidae) in corn and *Dendrolimus punctatus* (Lepidoptera: Lasiocampidae) on pines (Hussey and Tinsley, 1981). In addition, *B. bassiana* has been shown to have some pathogenicity in the laboratory against several target and non-target organisms, such as *Alphitobius diaperinus* (Coleoptera: Tenebrionidae) (Rohrlich et al., 2018) and *Chrysoperla externa* (Neuroptera: Chrysopidae) (Amorim et al., 2005). The B4 strains screened in this study showed high virulence against each developmental stage of *B. dorsalis* and have potential for field application in the future biological control of *B. dorsalis.*

The B4 strain caused the highest mortality to adults, followed by pupae and larvae, while eggs were largely unsusceptible. Similar results have been reported for *Bactrocera zonata* (Mahmoud, 2009; Hussein et al., 2018; Usman et al., 2021) and *B. cucurbitae* (Hamzah et al., 2021). However, the virulence of larvae and pupae to *B. bassiana* in previous studies differed from that described here. In *B. zonata* (Gul et al., 2015; Hussein et al., 2018; Usman et al., 2021) and *C. capitata* (Soliman et al., 2020) larvae were more susceptible than pupae. Moreover, Usman et al. (2021) and Wakil et al. (2022) found that *B. dorsalis* pupae were more susceptible than larvae, which does not align with our current findings. The contrasting results may be due to differences between the strains of *B. bassiana*, the method of application, and the age of the pupae (Rizvi et al., 2009; Beris et al., 2013; Gul et al., 2015; Soliman et al., 2020; Shaurub, 2022). We focused on one- to two-day-old pupae, whereas previous research showed that the older the pupae, the less susceptible they are to EPF (Poprawski et al., 1985; Hussein et al., 2018). This may be related to the softer cuticle of young pupae, which allows the fungus to penetrate the epidermis more easily (Mora et al., 2018).

Temperature and humidity are the most important environmental factors affecting the growth and virulence of *B. bassiana* (Dorschner et al., 1991; Shaurub, 2022). Our results are consistent with Pan et al. (2008), who found that 25°C and 90–100% RH were the optimum temperature and humidity for spore germination and colony growth. The virulence of *B. bassiana* to each stage of *B. dorsalis* was optimized under these conditions. The soil moisture content is critical for the larval pupation and pupal emergence (Ekesi et al., 2003; Quesada-Moraga et al., 2006). The virulence of *B. bassiana* is the highest at a soil water content of 50–70%, and *B. bassiana* may grow best under these conditions. The growth and infestation of *B. bassiana* are influenced by various environmental factors. Therefore, spraying chemicals in cloudy weather or after rain is conducive to *B. bassiana* germinating to achieve optimal control effects (Pan et al., 2014).

Combining chemical agents with EPF is a promising pest control option to minimize harmful chemical effects (Karthiba et al., 2010; Pelizza et al., 2018). Mixing *B. bassiana* and deltamethrin was effective in controlling *Hyalomma anatolicum* (Acari: Ixodidae) (Sun et al., 2011). The inhibitory effect of beta cypermethrin on spore formation and conidial germination of *B. bassiana* was the lowest at the recommended concentration of 10%, and the mortality rate of *Phauda flammans* (Lepidoptera: Phaudidae) was significantly increased when *B. bassiana* was mixed with beta cypermethrin at the recommended concentration of 10% (Chen et al., 2021). However, in the current study, 1.8% avermectin EC showed high inhibition of the B4 strain, while the other agents had a relatively small effect. This indicates that 1.8% avermectin EC should not be mixed with the B4 strain or applied concurrently in specific field applications. However, further research is needed on specific concentrations that maximize the efficacy of chemicals with *B. bassiana* against *B. dorsalis*.

The combined use of EPF and arthropod natural enemies in an integrated pest management strategy has been previously explored (González-Mas et al., 2019). Several studies have examined the safety and efficacy of combining EPF and other biocontrol components, e.g., predators, parasitoids, and nematodes (Roy and Pell, 2000; Acevedo et al., 2007; Labbé et al., 2009; Ansari et al., 2010; Martins et al., 2014). Gadhave et al. (2016) indicated that *B. bassiana* does not affect the parasitoid species associated with pea leafminer. Similar results were obtained for *Myzus persicae* (Hemiptera: Aphididae) and its parasite *Aphidius colemani* (Hymenoptera: Braconidae) (Jaber and Araj, 2018). However, we found that the B4 strain had a constraining effect on the growth, development, and reproduction of *A. cucumeris* and *A. japonicus* populations. This is consistent with the findings by Gao et al. (2022) that *B. bassiana* was highly pathogenic to non-target *Spodoptera frugiperda* (Lepidoptera: Noctuidae). The host range of *B. bassiana* is very wide, so consideration of its effects on non-target species is an important factor when using EPF in integrated pest management (Rohrlich et al., 2018). Therefore, it is important to avoid releasing *A. cucumeris* and *A. japonicus* at the same time as applying *B. bassiana* in the field. Furthermore, the relatively low diversity within agroecosystems may influence natural enemies within (Schmitz, 2007; Cappa et al., 2022). Field application of the B4 strain should therefore consider not only the natural enemies of *B. dorsalis*, but also the protection of the natural enemies of other pest species to prevent significant losses within agroecosystems, indirectly exacerbating the environmental pollution caused by chemical pesticides.

## 5 Conclusion

B4 controls several *B. dorsalis* life stages, particularly under optimal environmental conditions for growth and virulence. We also provide an empirical basis for combining the B4 strain with chemicals and natural enemies of *B. dorsalis* for improved virulence in the field. In summary, we provide an important practical base for the development of *B. bassiana* formulations to aid in the control of *B. dorsalis* in the field.

## Data availability statement

The original contributions presented in the study are included in the article/supplementary material, further inquiries can be directed to the corresponding author.

## Ethics statement

The manuscript presents research on animals that do not require ethical approval for their study.

## Author contributions

X-lL: Conceptualization, Data curation, Software, Validation, Visualization, Writing – original draft, Writing – review & editing. J-jZ: Conceptualization, Data curation, Investigation, Software, Validation, Writing – review & editing. D-dL: Data curation, Validation, Writing – review & editing. X-yC: Data curation, Software, Writing – review & editing. Y-xQ: Data curation, Funding acquisition, Writing – review & editing. Y-yL: Conceptualization, Funding acquisition, Project administration, Writing – review & editing.
